# Advances in the Treatment of Polycythemia Vera: Trends in Disease Management

**DOI:** 10.7759/cureus.14193

**Published:** 2021-03-30

**Authors:** Yajur Arya, Arshi Syal, Monica Gupta, Saurabh Gaba

**Affiliations:** 1 Internal Medicine, Government Medical College and Hospital, Chandigarh, Chandigarh, IND

**Keywords:** cytoreduction, polycythemia vera, hydroxyurea, ruxolitinib, interferon, treatment

## Abstract

Treatment modalities for polycythemia vera (PV) have evolved over time. Phlebotomy and low-dose aspirin suffice in low-risk patients, but cytoreductive therapies are indicated in all high-risk patients (age ≥ 65 years or those with a history of PV-related thrombotic event) and may be considered for low-risk patients with progressively increasing splenomegaly, progressively increasing leucocyte and platelet counts, and for those who do not tolerate phlebotomy. Hydroxyurea/hydroxycarbamide or interferons can be used as first-line drugs. Hydroxyurea may not be tolerated by some patients, and it also carries risk of myelosuppression. Interferon alfa is especially useful for PV symptoms, and the newer preparation, ropeginterferon alfa-2b, has lesser incidence of flu-like reactions. Ruxolitinib reduces the JAK2V617F mutation burden and is used as a second-line drug. Anagrelide reduces platelet production and can be used in conjunction with hydroxyurea in patients with excessive thrombocytosis. The alkylating agent, busulfan, can also be used as a last resort in patients with a limited life expectancy. Prospective future treatments include givinostat, a histone deacetylase inhibitor, and idasanutlin, a murine double minute 2 antagonist.

## Introduction and background

Polycythemia vera (PV), one of the myeloproliferative malignancies, is characterized by clonal proliferation of hematopoietic cells, chiefly erythrocytes. The pathophysiology of myeloproliferative disorders is based on the presence of certain genetic mutations. A majority of the cases of PV are accompanied by Janus kinase-2 (JAK-2) mutations, mainly the JAK2V617F mutation [1]. This results in a nucleotide switch and, in turn, a qualitative genetic abnormality resulting in replacement of valine by phenylalanine at codon 617 on chromosome 9 [2]. The identification of this mutation has led to the discovery of novel treatment options, which have revolutionized the management of PV. The clinical spectrum of PV is vast from being detected incidentally to a full blown picture with typical signs and symptoms chiefly pertaining to increased red blood cell (RBC) number and mass, thereby leading to increased blood viscosity. This predisposes to thrombotic complications along with hemorrhagic complications due to production of dysfunctional platelets. There is increased synthesis of various cytokines, which leads constitutional features such as fever, pruritus, weight loss, and anorexia. These symptoms produce significant morbidity and loss of productivity [3]. The cornerstone of management includes reducing thrombotic events, managing constitutional symptoms, and halting the progression of malignancy while minimizing side effects associated with various therapeutic modalities [4].

## Review

Methods

A literature search was performed on four biomedical electronic search engines (PubMed, Scopus, Google Scholar, and ScienceDirect). The search strings used were “polycythemia vera” AND “treatment” OR “updates” OR “advances” OR “management” to identify relevant studies. A narrative review has been prepared using review articles and clinical trials to reflect the current footing of various drugs and possible future treatments.

Discussion

Treatment strategies in PV have evolved in the past few years. Before the introduction of various specific cytoreductive treatment modalities, the major aim of management was to reduce thrombotic and hemorrhagic events to prevent complications and to monitor for development of sequelae such as acute myeloid leukemia and myelofibrosis [5]. The cytoreductive therapies are indicated in high-risk patients (age ≥ 65 years or those with a history of PV-related thrombotic event) and may be considered for patients with progressively increasing splenomegaly, progressively increasing leucocyte and platelet counts, and for patients who do not tolerate phlebotomy [6].

Phlebotomy and Low-Dose Aspirin

The simultaneous use of low-dose aspirin (81 mg/day) and phlebotomy to achieve a target hematocrit of less than 45% has been widely followed [7]. This combination serves as a first-line treatment option for patients with low-risk disease and can be used in addition to cytoreductive therapy in patients belonging to the high-risk group [8]. Earlier studies reported an increased risk of thrombosis in PV patients who received more than three phlebotomies per year in conjunction with hydroxyurea [9]. However, recent studies have refuted this claim, highlighting the importance of maintaining a hematocrit of less than 45% for adequate disease management [8]. Low-dose aspirin has been shown to significantly decrease the risk of mortality from cardiovascular complications without significantly increasing bleeding incidents [7]. Large-scale estimation of the risks and benefits associated with aspirin use in PV and consensus for its recommendation is currently lacking.

Hydroxyurea/Hydroxycarbamide

This well-known drug is one of the most widely used cytoreductive agents with wide application in the management of PV. It acts by inhibiting the enzyme ribonucleoside reductase and thus inhibits deoxyribose nucleic acid (DNA) synthesis [10]. It is the first-line cytoreductive agent in patients with high-risk PV [11]. Its undesirable action is predominantly on the hematopoietic system, frequently leading to myelosuppression. Intolerance to hydroxyurea is fairly common, probably due to adverse effects like mucositis, ulceration, dermatitis, and development of skin cancer [12]. Alvarez-Larrán et al. documented the development of resistance to hydroxyurea therapy. This was associated with an increased risk of mortality (5.6 fold) and transformation to acute leukemia (6.8 fold) in patients developing resistance [13]. Hence, additional studies are needed to study the phenomenon of resistance.

Ruxolitinib

Ruxolitinib, a promising novel agent in the management of PV, directly targets the primary pathogenetic mutation implicated in PV (JAK2V617F). It was approved by the Food and Drug Administration (FDA) in 2014. Focus was shifted to this drug in view of emerging resistance to and side effect profile of hydroxyurea. Verstovsek et al. reported excellent clinical efficacy of ruxolitinib in their patient’s refractory or intolerant to hydroxyurea. Not only were the symptoms ameliorated with this novel agent, but also the hematocrit and complete blood counts normalized, and a resolution of splenomegaly was also noted [14]. Ruxolitinib was found to improve the clinical picture better than the previously available therapeutic modalities in the RESPONSE trial as well [15]. Its effect at the molecular level was prominent with reduction of JAK2V617F mutation burden in PV patients [16]. Ruxolitinib was also shown to be effective in hydroxyurea-resistant/intolerant patients without splenomegaly [17]. This drug is not free of adverse effects, which include anemia, thrombocytopenia, herpes zoster reactivation, and development of non-melanomatous skin cancers. Vaccination to prevent reactivation of zoster is thus advocated prior to treatment [15,18]. Evidence-based medicine has revealed the favorable potential of ruxolitinib in PV, with alleviation of symptoms and improved patient-reported outcomes and quality of life [19]. A study to uncover the potential of momelotinib (a similar JAK inhibitor) in PV was also conducted but terminated due to low efficacy [20]. Currently, use of ruxolitinib is limited to PV resistant to hydroxyurea and in cases of intolerance to hydroxyurea [6].

Anagrelide

Anagrelide is an antithrombotic and platelet-reducing agent that is also being used to treat thrombocythemia associated with other myeloproliferative diseases. It is an anti-platelet drug with co-incidental cytoreductive action. It was originally developed to be used as an anticoagulant, but it surprisingly showed potential in essential thrombocythemia [21]. However, its use in PV is limited to where reduction in platelet counts is required. There is no consensus recommendation for its use in PV, although some clinicians use it in patients with platelet counts of >1500 x 10^9^/L [22]. Studies have shown that hydroxyurea has a better effect on JAK2V617F mutation-harboring cells as compared to anagrelide [23]. Thus, use of anagrelide monotherapy in PV is not recommended, and commencement of this drug along with existing therapy should be considered on a case-to-case basis.

Interferons

Interferon alpha limits RBC proliferation in PV. It remains one of the first-line treatment options for PV, especially in pregnant females [24]. Its clinical efficacy has been reported to be excellent, similar to that of hydroxyurea [25]. Interferon alpha improves the symptom profile in PV patients with control of pruritus, paresthesia, and erythromelalgia [26]. Studies have shown that early initiation of therapy with interferons can lead to sustained remission in patients with myeloproliferative disorders [27,28]. However, the use of interferons is not devoid of adverse effects, the common ones being flu-like symptoms, back and joint pain. Some of the serious toxicities include hematological changes like anemia and lymphopenia, and interferon therapy has also been linked to occurrence of autoimmune disorders [26]. Another drawback of interferon use is the tedious dosing regimen (thrice weekly injections, approximately three million units per dose). Pegylated interferons were introduced to overcome this major disadvantage, and they also offer a better side effect profile [29]. Studies have shown that low-dose pegylated interferon alpha 2 has an even better efficacy when given in combination with ruxolitinib, characterized by improvement in cell counts and the bone marrow picture, reduced JAK2V617F mutation burden, and adequate amelioration of symptoms [30]. The most commonly reported adverse effects with its use include malaise, myalgias, nausea, vomiting, and depressive episodes [29]. In an attempt to counteract these undesirable effects and retain the efficacy of this drug, a newer formulation has been devised, known as ropeginterferon alfa-2b.

Ropeginterferon Alfa-2b

Ropeginterferon alfa-2b is a novel interferon with an increased half-life, allowing for a simplified dosing regimen, a major limiting factor of traditional interferons [29]. It has an anti-proliferative effect on stem cells having the JAK2 mutation [31]. It has been approved for use in PV in patients without symptomatic splenomegaly and as monotherapy as well [32]. Many clinical trials have been performed to investigate the efficacy of ropeginterferon alfa-2b, the most important ones being PROUD-PV and its extension CONTINUATION-PV [33]. These trials compared ropeginterferon alfa-2b with hydroxyurea. Although significant clinical efficacy in terms of reduction of splenomegaly was not demonstrated, complete hematological response was observed in 53% of the participants. This response kept on increasing with continued administration [29]. Ropeginterferon has also been studied in combination with phlebotomy and aspirin for management of low-risk PV patients and was found to be better than the combination of phlebotomy and aspirin [34]. It is noteworthy to mention that the adverse effects associated with this drug include a drop in leukocyte and platelet counts as well as flu-like symptoms, malaise, arthralgias, and increments in aminotransferases. Thus, ropeginterferon alfa-2b could prove to be a game changer in the management of PV in the future.

Busulfan

The cytotoxic drug, busulfan, is used in cases of PV having a short-life expectancy along with resistance or intolerance to first-line (hydroxyurea and interferons) and second-line agents (ruxolitinib) [6]. It is a cheap and potent alkylating agent that non-specifically inhibits the cell cycle. The adverse effects include cytopenias, pulmonary fibrosis, seizures, hepatic veno-occlusive disease, and leukemic transformation. Kuriakose et al. administered bulsulfan to six patients with PV refractory to all other cytoreductive therapies and achieved complete hematological response in all of the them within three months. The median duration of therapy was 56.5 months [35]. None of the patients developed leukemia, while only one developed thrombocytopenia. Alvarez-Larrán et al. investigated its role in PV and essential thrombocythemia, and they documented complete hematological response in 83% of their study population after a median duration of 203 days [36]. The therapy had to be discontinued in 30% of all the recipients due to development of bone marrow suppression, and one patient developed acute leukemia.

Histone Deacetylase Inhibitors

Histone deacetylase (HDAC) is an enzyme that downregulates the expression of tumor suppressor genes. Hence, HDAC inhibitors are potentially anti-oncogenic in nature. The drug givinostat belongs to this category. It prevents the synthesis of mutated JAK2 protein without having any effect on the wild-type protein [37]. A study revealed the tremendous potential of this novel drug in PV. Both complete and partial hematological recoveries were documented in addition to improvement in splenomegaly and pruritus. Some patients remained in remission even with discontinuation of phlebotomies as adjunct to HDAC inhibitors. The mutation burden was also found to be decreased [38]. A synergistic effect was observed when givinostat was combined with hydroxycarbamide, with better control of pruritus and improvement in cell counts [39]. Important adverse effects include diarrhea, thrombocytopenia, and increased serum creatinine levels [40]. Large clinical trials are needed to evaluate the efficacy and safety of this novel agent. Nevertheless, givinostat seems to be a promising therapeutic modality for PV in the near future.

Murine Double Minute 2 Antagonists

Murine double minute 2 (MDM2) antagonist is a protein that downregulates the major tumor suppressor gene p53, inhibiting its transcription and simultaneously enhancing its destruction [41,42]. Considering the anti-tumor potential of p53 in the biological system, this alteration leads to enhanced oncogenesis. Thus, MDM2 antagonists potentially serve as p53 upregulators and have a tumor-suppressing effect. Two important drugs belong to this class, nutlin-3 and idasanutlin. Nutlin-3 was combined with pegylated interferon 2 alpha in culture media, which reduced the number of PV CD34+ hematopoietic stem cells [43]. RG7112 is another drug closely related to nutlin-3 with even better potency. Its efficacy was evaluated by Lu et al. [43]. They reported that combination treatment with RG7112 and pegylated interferon 2 alpha decreases the hematopoietic stem cell population in PV significantly [44]. Mascarenhas et al. reported an excellent clinical efficiency of idasanutlin therapy in their study. The important side effects of this therapy included diarrhea, fatigue, and nausea. Because of the promising results shown in initial studies, a global phase two trial of idasanutlin therapy for hydroxyurea-resistant/intolerant PV patients is currently underway [44]. The current role of various cytoreductive drugs has been summarized in Table 1.

**Table 1:** The current role of various cytoreductive drugs in the treatment of polycythemia vera

Drug	Role
Hyroxyurea	First line or second line (when interferons are used as first line)
Interferons	First line or second line (when hydroxyurea is used as first line)
Ruxolitinib	Second line or third line
Anagrelide	Third line (only in combination with hydroxyurea)
Busulfan	Third line (in patients with limited life expectancy)
Givinostat	Not currently approved
Idasanutlin	Not currently approved

## Conclusions

Treatment options for PV have evolved with time. Phlebotomy with low-dose aspirin forms the standard of care for low-risk patients, and cytoreductive therapies are indicated in high-risk cases and some low-risk cases. Hydroxyurea is currently the most widely prescribed drug. Recent studies have established the safety and efficacy of ruxolitinib and ropeginterferon alfa-2b in the management of PV. These are especially important in a scenario of emerging resistance and intolerance to hydroxyurea. Anagrelide and busulfan are third-line drugs. Newer modalities like MDM2 inhibitors and HDAC inhibitors have been found to be effective in certain pilot studies, and more data are needed before they form a part of patient care.
